# A Co-Designed Social Media Intervention to Satisfy Information Needs and Improve Outcomes of Patients With Chronic Kidney Disease: Longitudinal Study

**DOI:** 10.2196/13207

**Published:** 2020-01-27

**Authors:** Cristina Mihaela Vasilica, Alison Brettle, Paula Ormandy

**Affiliations:** 1 The University of Salford School of Health and Society Salford United Kingdom

**Keywords:** social media, patients outcomes, long term condition, chronic kidney disease, self-efficacy, patients information needs, co-design

## Abstract

**Background:**

The number of people living with a long-term condition is increasing worldwide. Social media offers opportunities for patients to exchange information and experiences with others with the same condition, potentially leading to better self-management and improved patient outcomes, at minimal costs to health service providers.

**Objective:**

This paper describes how an online network with a range of social media platforms was created, with the help of a group of patients with chronic kidney disease and specialist professionals. The project considered whether information needs and health-related and social outcomes were met.

**Methods:**

We performed a longitudinal in-depth evaluation of the creation of the moderated network, observation of the use of the platforms, self-efficacy surveys (at baseline and 6 months), and semistructured interviews (at baseline and 6 months).

**Results:**

A total of 15 patients and professionals participated in the co-design of the network (hub), which was initially launched with 50 patients. Several platforms were needed to engage patients at different levels and encourage generation of information, with the support of moderators. In addition, 14 separate patients participated in the evaluation. Satisfaction of information needs through social engagement improved self-efficacy (n=13) with better self-care and management of illness. Social outcomes included seeking employment and an increase in social capital.

**Conclusions:**

An online network (hub) with several social media platforms helped patients with chronic kidney disease manage their condition. Careful co-designing with users resulted in a sustainable network with wider applicability across health and social care.

## Introduction

The increase in long-term conditions is seen as the greatest challenge faced by health systems globally [1], with one in three adults affected by multiple chronic conditions [2]. In England alone, the number of long-term conditions is estimated to reach 2.9 million in 2018, accounting for 70% of total health and social care spending [3]. From international to local levels, there is an increased focus on innovation and patient-centered and preventative care [3-7] including patient engagement with electronic health systems [8,9].

Information provision for patients often occurs as a result of a problem or symptom as well as dependence on the specific needs of the patients. “Information need is a recognition that your knowledge is inadequate to satisfy a goal that you have, within the context/situation that you find yourself at a specific point in the time” [10]. Information behavior is the totality of human behavior concerned with channels of information that involves information seeking and use [11]. Patients engage in information behavior activities at different stages of their illness [12,13].

Research acknowledges that effective provision of information is a determinant in helping people self-manage their own illness [12], which then has the potential to improve self-care, health behavior, and quality of life [13,14]. Yet, satisfying the information needs of patients remains a challenge [10,15]. Patients with chronic kidney disease may not recognize that they need information [10], or the information they need to alleviate the uncertainty of the condition is not available [16]. Nevertheless, they are interested in talking to each other to gain knowledge about the condition and access peer support [10,16] and while on dialysis, they develop new relationships with clinical staff and the dialysis patient community [16]. Recent systematic reviews of online peer-to-peer communities suggest that they provide a supportive space for daily self-care related to chronic illness and a valued space to strengthen social ties and exchange knowledge that extends beyond the illness and medical care [17,18].

Social media provides opportunities for user-generated peer content, which embraces knowledge transfer (eg, advice, information, and resources) and support (eg, companionship) to address patient engagement, access to information, and positive outcomes. This model of information generation moves away from clinician-led information provision to patient-generated information in order to support patients’ needs and positively influence patient self-management [19]. Social media allows access to information and support at a time and context that suits the patient. However, the inconsistency and quality of information shared via social media networks poses significant challenges [20], and the variety of social media platforms (eg, Facebook, Twitter, blogs, and forums) and their different audiences adds an additional challenge to those conducting research in terms of deciding which platforms to choose.

Research suggests that social media can be used in long-term conditions to exchange information and trigger positive outcomes [21]. However, an understanding of how kidney patients actually engage with these platforms and the resulting information generation and outcomes are lacking. This is important to effectively exploit the potential of social media for meeting the information and supporting the needs of patients with long-term conditions.

This study therefore aimed to use a variety of linked social media (a hub) to encourage patients with chronic kidney disease in one area of the United Kingdom to generate (post) information and respond to the contributions of others. A social media hub was co-designed with patients and then evaluated to determine whether it met patients’ information needs and improved health and social outcomes.

## Methods

### Approach

The project used a realist [22], longitudinal, mixed methods approach over two phases: (1) design and training, and (2) longitudinal evaluation.

Ethics approval was obtained from the University of Salford, who hosted the study and the system used within the UK Health service (NHS Research Ethics) prior to recruiting patients in the longitudinal study. All participants involved in the longitudinal evaluation study provided written consent.

### Setting and Sample

**Figure 1:**
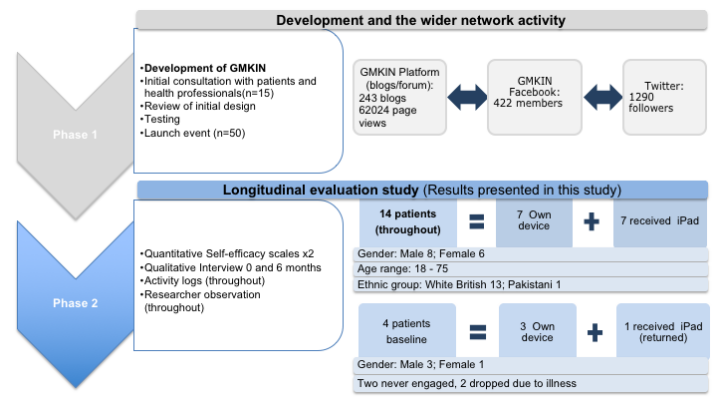
Overview of the sample. GMKIN: Greater Manchester Kidney Information Network.

The study took place in the North West of England, following meetings with the local Kidney Patient Association, patients, and carers recruited via local health care professionals from a large teaching hospital. A total of 15 users (patients, carers, health practitioners, and researchers) engaged in the co-design of the social media hub (online network). A launch event was held with 50 patients to provide training, and the majority signed up to join the hub and Facebook group. For the longitudinal evaluation, 17 separate patients with chronic kidney disease and 1 carer were recruited via the local Kidney Patients Association, Facebook, and word of mouth. Patients were eligible for inclusion if they were aged over 18 years, had chronic kidney disease (predialysis, hemodialysis, peritoneal dialysis, or transplant), were recommended by a health care professional, could provide written informed consent, and could read and write English. A theoretical sampling approach was used to ensure a mix of ages, gender, and stages of kidney disease and to ensure that this sample group did not overlap with those involved in the design. The carer was included because he used the Greater Manchester Kidney Information Network (GMKIN) on behalf of his non–English-speaking mother. To maximize inclusion, patients with no access to technology (n=7) received an iPad and additional training to facilitate participation. Four patients dropped out (two did not engage at all and two could not take part in the final interview due to illness). Over the period of the evaluation, health professionals and patients were free to join and use the hub, contributing to hub activity, in general. It is not possible to calculate the number of users of the hub over the evaluation period, but activity in the hub in this time frame is presented in Figure 1.

### Phase One: Design and Training

#### Hub Design

Meetings with the Kidney Patient Association, patients, and carers identified a clear need to find innovative ways to enable patient access to health information and support. Findings from previous research [23] were combined with the user engagement and information needs theory [10,24,25] to inform a user-centered design (UCD) process [26], led by the lead researcher (CV) and involving a three-stage iteration [27]:

1. Initial consultation with patients and health professionals, reviewing social media platforms, and a potential hub to support patient-centered care and self-management
2. Presentation of hub and discussion on color scheme, usability, and accessibility (create accounts, write and share posts, add comments) and integration with social other social media platforms
3. Platform testing and virtual meeting (using Facebook) to refine the hub before releasing it to the public

The hub was named GMKIN and incorporated social media platforms with active users (Facebook), advocacy (Twitter), blogging, and a forum (Multimedia Appendix 1). The Facebook group was initially open (setting for anybody to see group members and their posts) to attract new membership and then closed (setting for only members of the group) to protect the confidentiality of the information posted. A launch event provided training and registration.

#### Moderation

In line with most existing Web platforms that moderate posts and comments before publishing [25], the GMKIN was moderated by a manager (CV) and a patient (P1) as follows:

- GMKIN Platform: Blogs (patients’ stories) were screened prior to publication on the hub. Those with potential health risks were referred to a multidisciplinary group of health care professionals who signposted patients to a relevant service. Comments posted relating to blogs were approved by either the moderators or the author of the blog.
- GMKIN Facebook: Moderators screened each post following update notifications.

The manager actively encouraged and motivated members to take an active role in the GMKIN to influence community growth and foster underlying psychological bonds. Community commitment and relationship building were facilitated by using the principles of social capital (bonding, bridging, and linking) [28,29], which involved creating an identity based on local interest (North West), shared values, interests, and goals, reaching broader audiences with an informal tone and humor.

### Phase Two: Longitudinal Evaluation

Phase two aimed to explore patients’ engagement with the hub, information generation, and health and social outcomes.

#### Data Collection

Quantitative and qualitative data collection tools (Table 1) were used to obtain multiple perspectives, capture user experience. Interviews were conducted at a location convenient for the participant (hospital, home, or university setting), lasted 1-2 hours, and were audiorecorded.

This created an in-depth rich triangulated data set from which to draw the conclusions.

**Table 1:** Data collection methods.

Data collection method	Tool	Purpose
Quantitative Self-efficacy scales (two scales; at 0 and 6 months)	General Perceived Self-Efficacy Scale [30] Self-Efficacy for Managing Chronic Disease 6-Item Scale [31]	Used as a barometer to identify the difference in self-efficacy after becoming involved in the GMKIN
Activity data/researcher observation (throughout)	Complement weekly logs by collecting activity data (eg, inbuilt analytics within the website software [WordPress plugin]) and monitoring engagement via manual monitoring of Facebook and twitter	Create individual profile of monthly activity and interactions and identify platform usage
Qualitative interview at 0 and 6 months	Semi-structured interview	Understand the context of each patient entering the study and gain in-depth knowledge on key themes explored: levels of engagement, role of each platform, information needs, and outcomes
Patient logs/blogs (throughout)	Blogs posted on the platforms (using WordPress). Self-reported weekly activity logs, using Google docs, of GMKIN activity to capture rich descriptions of “*real life”* [32]: 11 across space and time	Understand levels of engagement, what works, and why

#### Data Analysis and Synthesis

The data were collected, analyzed, and synthesized by the GMKIN Manager and lead researcher (CV). Methods to reduce bias included multiple methods of data collection (eg, checking interview statements against patient activity) and checking coding of interview transcripts with other members of research team.

#### Quantitative Data

The quantitative data from the self-efficacy scales were analyzed to determine the mean score of the six items by using descriptive statistics in Microsoft Excel (Microsoft Corporation, Redmond, Washington). The higher the score, the higher the indication of self-efficacy. A *t* test was performed to determine if the results were statistically significant between baseline and follow-up. As the small sample size was unsuitable for any further statistical test, the Self-Efficacy for Managing Chronic Disease 6-Item Scale (CSE) and General Perceived Self-Efficacy Scale (GSE) scores were used to as a barometer to inform the discussion within the context of patient interviews, rather than demonstrate effectiveness.

#### Activity Data/Patient Logs/Blogs

Activity data were collected using a WordPress plug-in for patient blogs and manual observation of Facebook posts and Twitter feeds.

#### Interviews

Baseline and 6-month data were analyzed using a case and thematic analysis to describe and map conceptual findings [32] in relation to context mechanisms and outcomes [22]. Baseline data were used to create a framework, which created a starting point for analysis of the 6-month data.

#### Data Synthesis

A framework (matrix) approach was used to synthesize cross-sectional descriptive qualitative data using the following steps:

- Matrix development from themes identified from a literature review [33]
- Theming and mapping the interviews against the framework. The matrix was expanded to include the new themes.
- Data were compared and contrasted across individual cases to explore contextual factors and patient outcomes.
- Activity data were used to create individual case engagement logs.

## Results

### Sample

Figure 1 provides an overview of the participants and network activity throughout phases 1 and 2. A total of 15 patients and health professionals were involved in the hub design; 50 patients attended the launch event (phase 1), and 14 separate participants are reported in the longitudinal evaluation (phase 2). Participants in the evaluation phase spanned a range of ages, with a comparable number of male and female participants, and included people at different stages of chronic kidney disease, receiving different treatments, and a carer.

### Quantitative Results for Self-Efficacy

Table 2 shows that 13 of 14 patients indicated an increase in self-efficacy at least for one of the instruments from baseline to 6 months, with one reporting a decrease in self-efficacy. It is worth noting that patients who reported that they were had depression before or at the point of joining the GMKIN (P1, P5, and P12) increased their self-efficacy across all domains within the first 6 months.

Activity data revealed three engagement roles: influencers, conversationalists, and browsers [25]. There was one *influencer* who described engagement as a facilitator of meaningful relationships among users through light discussions, sociability, and support of prospective leaders. Four *conversationalists* were crucial to sustaining conversations and contributed to content creation and provision of feedback, while nine *browsers* read and collected information and preferred to engage in this way, because they perceived that they did not have enough knowledge or experience to share. An important finding is that patients from all different roles of engagement (influencers, conversationalists, and browsers) benefited equally in terms of self-efficacy. This was further reinforced by the qualitative findings (see below).

**Table 2:** Self-efficacy trend for Self-Efficacy for Managing Chronic Disease 6-Item Scale and General Perceived Self-Efficacy Scale.

Patient	GMKIN^a^ role	iPad access	Age group	Modality	Gender	CSE^b^			GSE^c^	
						Change in self-efficacy	*P* value	Change in self-efficacy		*P* value
P1	Influencer	No	51-60	Transplant	Male	+^d^	0	+		.1
P2	Browser	Yes	51-60	Dialysis	Male	+	.46	+		.05
P4	Browser	No	<30	Carer	Male	+	.7	+		.1
P5	Browser	Yes	41-50	Dialysis	Male	+	.03	+		<.001
P12	Browser	Yes	<30	Predialysis	Female	+	.03	+		.002
P8	Browser	No	>61	Transplant	Male	+	.03	No change		Not applicable
P6	Browser	No	<30	Transplant	Male	+	.36	–^e^		.1
P14	Browser	Yes	31-40	Dialysis	Female	–	.14	+		.03
P13	Browser	Yes	51-60	Dialysis	Female	+	.08	–		.61
P7	Browser	No	>61	Predialysis	Female	–	.08	+		0
P3	Conversationalist	No	>61	Predialysis	Male	–	.47	+		.6
P11	Conversationalist	Yes	41-50	Dialysis	Male	–	.01	+		.06
P9	Conversationalist	Yes	31-40	Predialysis	Female	+	.79	–		.002
P10	Conversationalist	No	<30	Predialysis	Female	–	.04	–		.008

^a^GMKIN: Greater Manchester Kidney Information Network.

^b^CSE: Self-Efficacy for Managing Chronic Disease 6-Item Scale.

^c^GSE: General Perceived Self-Efficacy Scale.

^d^+: increase in self-efficacy.

^e^–: decrease in self-efficacy.

### Qualitative Findings (Information Needs and Outcomes)

Regardless of engagement role, patients’ information needs were satisfied and outcomes were improved as described below.

#### Information Provision to Satisfy Information Need

Patients reported in interviews that the patient-generated content shared in the form of blogs, posts, and tweets provided them with valuable information. Most respondents identified that through the GMKIN, they gained an understanding of the condition and its living implications:

I think it’s kind of triggered me to go and look at other things, and go and find out things, and I’ve learnt things that I didn’t know; like now, I know that there is, you can do home dialysis, which I never thought of.

P12

They learned about the different treatment options such as hemodialysis (HD), peritoneal dialysis (PD), home dialysis, and monitoring the progression of the disease:

To be quite honest with you I did not know the difference between HD and PD, I do now, but even before dialysis, that was it, I did not realise that were different forms of dialysis and this is something I picked up – just an example there are many things I picked up.

P3

Accessing information such as new clinical developments and people’s positive stories provided patients with the mechanisms to cope with the condition and give them hope for the future:

I have learnt this from a lot of people listening to their story that in relative terms my journey has not been easy but it’s been absolutely a piece of cake compared to what other people gone through and that’s made me realise perhaps my quality of life is better than what I was perceiving it beforehand.

P1

Patients explained how other people’s stories and updates helped them identify their own symptoms and develop management strategies:

The long-term effect of kidney disease is one of those things you don’t really know about...I read yesterday a link to her [patient] own blog, about anti-inflammatories, which I found quite interesting because I suffer a lot with sinus problems and I take anti-inflammatories which are bad for kidney...but they never tell you why, so I found out... I thought - I have been fine I have used them before so to take them again will not be too bad - but reading the post its best not to.

P10

The interviews demonstrated that patients were unaware of their information deficits. Some indicated that they had been told that the illness had progressed, but they only realized the implications after seeing other people’s symptoms on GMKIN:

I didn’t realise that the pains in my legs was due to my kidneys until somebody was writing it on.

P7

Importantly, engaging via the GMKIN gave patients confidence and a purpose, believing it could help others:

It has given me purpose… it has given me more focus. I have not allowed things like fatigue or lack of concentration to stop me. It has given me a motivation that was missing before that motivation is primarily to help others. I am feeling like genuinely helping other people, I think that is the essence of what we are like human beings this gives us the opportunity, GMKIN gives the opportunity to do it.

P1

Some patients, especially those new to chronic kidney disease, found the information overwhelming, but synthesized information pertinent to them to manage their condition better:

I’ll stop reading, then I try to put what they have said into my own mind to stop me doing certain things that I should not do to help to keep my kidney function.

P7

Discussing things like drugs or having discussions about the problems with getting supplies delivered…I really, really struggled and I’ve got to the part where I was talking to my partner and I was considering phoning you up and saying: No, I don’t want it.

P12

#### Health-Related Outcomes (Self-Efficacy, Self-Management, and Psychological Benefits)

The study revealed a positive impact on patient’s self-efficacy and self-management, which can be seen as a means toward achieving more quantifiable health outcomes such as improved kidney function. These are reported below, and in more detail, using patients’ stories in Multimedia Appendix 2.

Shared (vicarious) experiences and social persuasion contributed to an increase in self-efficacy, a key feature in the management of long-term conditions:

I think it helped me that he is going through so much and has dealt with the condition for such a long time and lived a positive normal life.

P9

Furthermore, patients who engaged with GMKIN reported better ability to self-manage the condition:

Watching and listening to what others are saying has helped me to sort my life out by managing my diet.

P7

Patients reported other psychological benefits including increased confidence and feeling generally better about themselves.

It’s almost been like a snowball effect, because once I’ve got over the kind of the shock and I dealt with things I recognised how the community on GMKIN was actually really helpful…and because I’d dealt with my issues I felt comfortable in getting involved in other things and that has increased my confidence.

P12

I don’t think I would have engaged with her before GMKIN I would not have felt confident enough in myself to be able to hold my own in a conversation with someone who clearly knows a lot about not just programme management but also renal problems and that is given me enormous satisfaction but again added to the boost in self-confidence.

P1

Accessing peer stories (although overwhelming for some) encouraged participants to make changes in their self-management to preserve their kidney function:

The condition and anything I can do to maintain a healthy lifestyle: eating the right food, drinking the right things, where to get travel insurance from, just general day to day things which is helps.

P8

#### Social Outcomes

In addition to health, GMKIN engagement demonstrated an impact in a range of social areas. A number of patients reported that they were now considering employment:

Entertaining the idea of getting some proper employment again and that would be an achievement. I never thought I do certainly giving the past 10 years in my life already didn’t think I could get to that point again.

P1

‘I applied for two jobs one with the kidney association, one of them I had to write things down, like an email.

P2

Patients who received an iPad acknowledged that it was enormously beneficial. One patient indicated that the iPad is his lifeline, which enabled him to be socially connected:

This is my lifeline [iPad]...this [iPad] is everything in one and then if you don’t want to watch anything at least you can look at someone else’s feelings read their things on GMKIN and if you want to know medical things, diet things everything is in one place, just look you don’t have to get bored.

P5

Another indicated that it enabled him to gain an interest in drawing:

I had never drawn so much then I had these past. Well I suppose since I’ve got the iPad, really. It’s influenced me a lot and my life and it’s helped me to sort of put away the troubles and stuff and just concentrate on the drawings.

P13

The affiliation with the community through bonding, bridging, and linking mechanisms such as light and friendly conversations (welcoming messages), social support, and the human touch (personal photos) suggested that GMKIN enabled trust, social camaraderie, friendship, and affection (or social capital) to be developed:

I just think it is amazing that people had the time to kind of develop something good for the condition, it is really nice that people are going on there and helping each other through, in this day and age when you read all this horrible stories and there isn’t much of social camaraderie really that people are taking the time and effort to support complete strangers through the condition.

P9

...bond in the sense, you know all this people, a bit of empathy and a bit of you know obviously banter and that it is good.

P3

## Discussion

### Principal Findings

**Figure 2:**
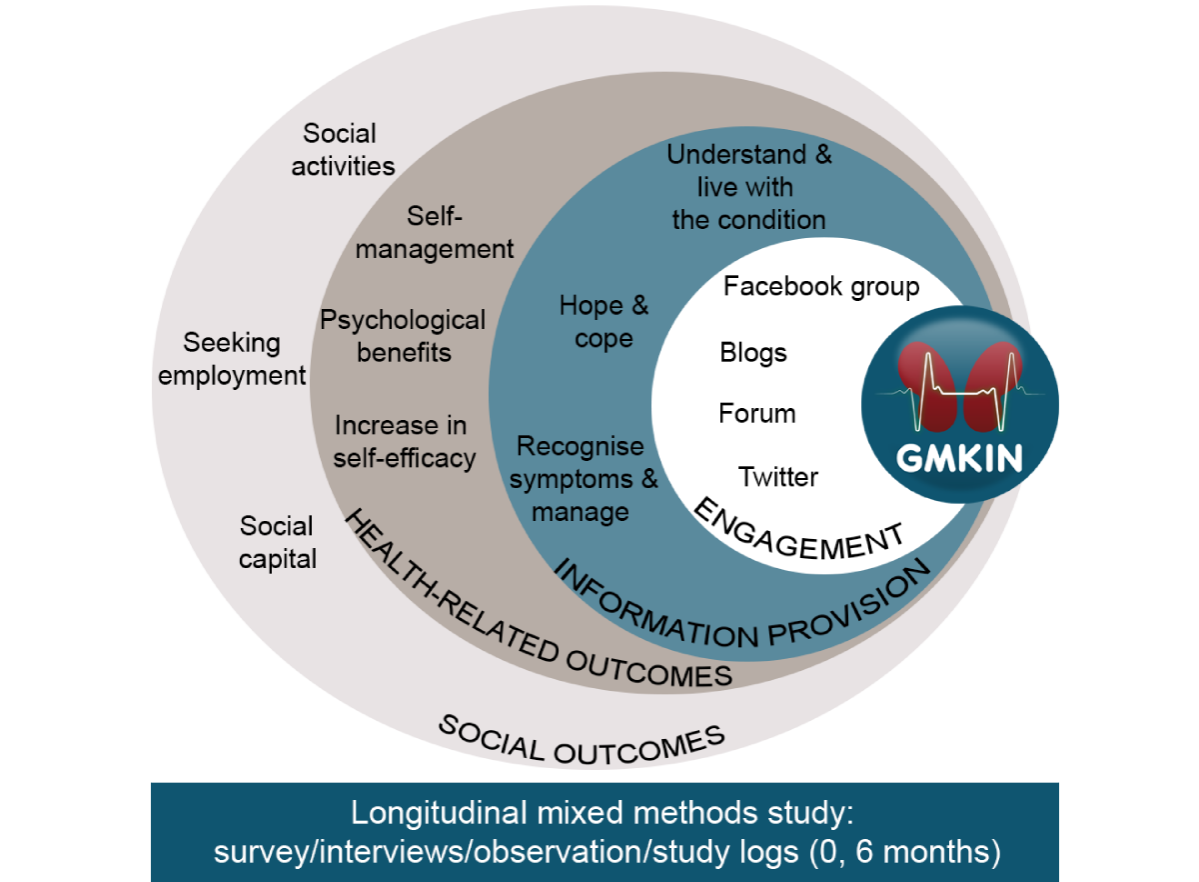
Overview of the findings. GMKIN: Greater Manchester Kidney Information Network.

This study used longitudinal data sets with a variety of quantitative and qualitative methods to enhance validity and rigor (Figure 1). It demonstrated that patient-generated information shared via social media contributes to satisfaction of information need and triggers positive health-related and social outcomes (Figure 2). These outcomes were achieved regardless of the way or extent to which patients engaged with the hub. This has wide-ranging and potential value in establishing similar hubs or online networks for others with long-term conditions and in contributing to national and international policy initiatives of promoting self-management [4-6].

The findings are in line with previous research in that social media contributes to an increase in self-efficacy [21]. Individuals draw on four different types of sources to discern self-efficacy: enactive mastery experience, vicarious experience, social persuasion, and physiological and emotional state [34]. Enactive mastery experience was linked to experiences in contributing to GMKIN and receiving positive feedback, which increased patient confidence in their ability to help other patients. Patients who engage in conversations with fellow community members are benefiting from accessing experiential information from peer stories [35]. Patients indicated that seeing other people’s stories had given them a new outlook on life, reducing negative perceptions of being different. The positive feedback posted by conversationalists on the GMKIN Platform and GMKIN Facebook encouraged patients to engage in posting and increased their self of worth and thus self-efficacy. Three patients with self-reported depression reported a statistically significant increase in at least one domain of self-efficacy of the GSE [30] or CSE [31]. This confirms previous research acknowledging the benefit of social media in increasing self-efficacy of patients with depression [36]. Bessière and colleagues [36] identified that using the Web for health information alone could increase depression, but using it to connect with friends will have a positive effect [36]. It is believed that the dual purpose of Facebook (friends and health) and the social capital developed through the network contributed to the positive effect [25]. In addition to the health-related outcomes, three patients intended to seek employment as a result of their involvement in GMKIN. This finding is unique to this study and is described as a social outcome of social media.

Although previous research highlighted that Facebook and blogs contribute to information generation [37-39], this study demonstrates that this information generation meets the previously identified information needs of kidney patients [10] such as living with the condition, symptoms, and expectations and self-management. These information needs are in line with information needs for other long-term conditions [12,40,41], suggesting wider potential application of the model.

This study also exposed that patients had unknown information deficits; for example, a participant indicated that he had not known about the different forms of treatment available until he read it on the network. Others measured themselves against patient stories and realized that they were not as ill as they had previously believed. By being part of the community and disclosing information, patients learned about their illness and how to self-manage it.

The GMKIN did not appear to work for everyone: Two patients dropped out because it was not fulfilling their needs and one remained in the sample but suggested in the interview that it did not work for him personally.

### Limitations

Although the study appeared to facilitate better health outcomes as a result of self-management, it is not clear whether this is directly linked to an increase in self-efficacy, as the quantitative findings are too small to be generalizable. However, the self-efficacy scores were used as a barometer to discuss self-efficacy further with patients and these qualitative data from interviews and direct observations of patients were in line with the quantitative scores.

### Wider Impact

Although not measured in the evaluation, one of the most significant outcomes of the project is a patient-led expansion to other UK regions. Patient-generated evidence of impact (Multimedia Appendix 2) is used to encourage other Kidney Patient Associations to join the GMKIN while retaining local autonomy. This has already been taken up in two other regions (Multimedia Appendix 3). Furthermore, responsibility for the network has been transferred to the patients, while the manager retains a supportive role and focuses on developing a new national model of patient-generated social media kidney disease support. More widely, knowledge generated through the GMKIN has contributed to the development of national guidance on the use of social media for patient and public engagement [42], and the theoretical learning regarding social media and engagement has influenced other initiatives linking patients/users and professionals via social media including antenatal care [43], rheumatic and musculoskeletal conditions (Multimedia Appendix 4), and prevention of the exploitation of young people [44].

### Conclusions

This mixed methods longitudinal study successfully co-designed and implemented a social media hub with patients and practitioners on the basis of the theory on engagement [24] and patient information needs for chronic kidney disease [10,23]. Patients within the online network used the hub to generate information about their long-term condition, which satisfied their information needs (including those they were unaware of), increased self-efficacy, and facilitated overall better health management and health and social outcomes. The positive outcomes achieved from this model has led to the development of a new national model of patient-generated information provision for those with kidney conditions via social media and influenced the theoretical development of other patient-focused social media initiatives and policies.

## Appendix

Multimedia Appendix 1 Gmkin link.

Multimedia Appendix 2 Patient voices.

Multimedia Appendix 3 Uptake of gmkin.

Multimedia Appendix 4 Uptake rheumatic and musculoskeletal conditions.

## Abbreviations

CSE: Self-Efficacy for Managing Chronic Disease 6-Item Scale
GMKIN: Greater Manchester Kidney Information Network
GSE: General Perceived Self-Efficacy Scale
HD: hemodialysis
PD: peritoneal dialysis
