# Supplementary material for: A Co-Designed Social Media Intervention to Satisfy Information Needs and Improve Outcomes of Patients With Chronic Kidney Disease: Longitudinal Study
Source: JMIR Form Res. 2020 Jan 27;4(1):e13207. doi: 10.2196/13207 (PMC7011121; doi:10.2196/13207)
Supplement: Multimedia Appendix 2 [file formative_v4i1e13207_app2.docx]

Multimedia Appendix 2

<https://youtu.be/nAC115vrmn8>).
